# Development of a non-destructive depth-selective quantification method for sub-percent carbon contents in steel using negative muon lifetime analysis

**DOI:** 10.1038/s41598-024-52255-5

**Published:** 2024-01-20

**Authors:** Kazuhiko Ninomiya, Michael Kenya Kubo, Makoto Inagaki, Go Yoshida, I-Huan Chiu, Takuto Kudo, Shunsuke Asari, Sawako Sentoku, Soshi Takeshita, Koichiro Shimomura, Naritoshi Kawamura, Patrick Strasser, Yasuhiro Miyake, Takashi U. Ito, Wataru Higemoto, Tsutomu Saito

**Affiliations:** 1 Institute for Radiation Sciences, Osaka University, 1-1, Machikaneyama, Toyonaka, Osaka 560-0043 Japan; 2 Graduate School of Science, Osaka University, 1-1, Machikaneyama, Toyonaka, Osaka 560-0043 Japan; 3 Division of Natural Sciences, International Christian University, 3-10-2, Osawa, Mitaka, Tokyo 181-8585 Japan; 4 Institute for Integrated Radiation and Nuclear Science, Kyoto University, Asashiro-Nishi, Kumatori, Osaka 590-0494 Japan; 5 Radiation Science Center, High Energy Accelerator Research Organization (KEK), 1-1, Oho, Tsukuba, Ibaraki 315-0801 Japan; 6 Muon Science Laboratory, Institute of Materials Structure Science, High Energy Accelerator Research Organization (KEK), 1-1, Oho, Tsukuba, Ibaraki 315-0801 Japan; 7 Advanced Science Research Center, Japan Atomic Energy Agency, 2-4, Shirakata, Tokai, Ibaraki 319-1195 Japan; 8 Department of Physics, Tokyo Institute of Technology, 2-12-1, Ookayama, Meguro, Tokyo 152-8550 Japan; 9 National Museum of Japanese History, 117 Jonai-Cho, Sakura, Chiba 285-8502 Japan

**Keywords:** Analytical chemistry, Nuclear chemistry, Exotic atoms and molecules

## Abstract

The amount of C in steel, which is critical in determining its properties, is strongly influenced by steel production technology. We propose a novel method of quantifying the bulk C content in steel non-destructively using muons. This revolutionary method may be used not only in the quality control of steel in production, but also in analyzing precious steel archaeological artifacts. A negatively charged muon forms an atomic system owing to its negative charge, and is finally absorbed into the nucleus or decays to an electron. The lifetimes of muons differ significantly, depending on whether they are trapped by Fe or C atoms, and identifying the elemental content at the muon stoppage position is possible via muon lifetime measurements. The relationship between the muon capture probabilities of C/Fe and the elemental content of C exhibits a good linearity, and the C content in the steel may be quantitatively determined via muon lifetime measurements. Furthermore, by controlling the incident energies of the muons, they may be stopped in each layer of a stacked sample consisting of three types of steel plates with thicknesses of 0.5 mm, and we successfully determined the C contents in the range 0.20–1.03 wt% depth-selectively, without sample destruction.

## Introduction

Steel is one of the most critical materials in use. It is the main structural component in almost all modern buildings and essential in cars and railroads. Steel mainly comprises Fe, with an atomic number of 26, and smaller amounts of other elements. With elements other than Fe, the properties of steel, such as hardness and toughness, may be controlled, and various types of steel may be produced according to their purposes. Among these minor elements, C is the most crucial. Steel commonly contains less than a few weight percent of C, but this significantly affects its properties. Generally, steel containing a relatively large amount of C of up to 1 wt% is hard but brittle, but that containing a small amount of C is relatively soft and tough. Thus, the C content is a critical parameter in understanding the properties of steel. Currently, a destructive chemical analysis involving the combustion of steel is used to analyze the amount of C in the steel. In this method, steel is decomposed at high temperatures and the emitted CO_2_ gas is quantified using infrared absorption^1^. Other methods of quantifying C using mass spectrometry^2,3^ have also been proposed. These destructive analytical methods are strongly affected by surface contamination by the air^4^, and thus, sample processing prior to analysis is delicate, and special equipment and techniques are required. However, non-destructive, accessible methods, such as X-ray fluorescence, may not be applied in steel analysis because the characteristic X-ray energy of C is too low. If the C content deep within bulk steel may be analyzed non-destructively and selectively without interference due to surface contamination, the method should be revolutionary in the quality control of steel, and numerous applications may arise. In this study, as a proposal for such an innovative analytical method, a muon beam, which is a quantum beam generated in an accelerator facility, was applied as a probe in non-destructively analyzing small amounts of C in steel.

A muon is an elementary particle that displays the same negative charge and spin as those of an electron and a mass approximately 200-fold larger than that of an electron. The muon decays to a high-energy electron (up to 50 MeV) and two neutrinos, with a lifetime of approximately 2.2 μs in a vacuum^5,6^. When a muon stops in a material, it is captured by an atom in the vicinity, i.e., the muon forms atomic orbits around the nucleus^7^. The muon capture probability of each atom depends on the elemental composition (in weight percentage) at the position of muon stoppage^8^. The captured muon immediately transitions into the muon 1 s atomic orbit (< 1 ps)^9^, emitting characteristic X-rays or Auger electrons. Remarkably, the energies of the characteristic X-rays produced by muon transitions (muonic X-rays) are approximately 200-fold larger than those of the characteristic X-rays produced by electron transitions. After reaching the muon 1 s atomic orbit, the muon is absorbed by the nucleus or decays to an electron. The rate of absorption into the nucleus depends on the atomic number (or more accurately, the isotope) of the atom capturing the muon in the atomic orbit^10^.

Muonic X-rays are used in the non-destructive identification of the elemental composition within a material^8^. This analytical method has been applied to various samples, such as archaeological artifacts^11–15^, extraterrestrial samples^16–18^, and Li-ion batteries^19^. However, since the sensitivity of each element is almost similar^8^, a low background measuring system is essential for the quantification of low concentration elements (< 1 wt%). In particular, detecting low concentration light elements is difficult because the low-energy muonic X-rays from light elements are interfered by the Compton scattering background signal of high-energy X-rays from heavy elements.

In this study, we propose a novel non-destructive sub-percent light element analytical method that measures the lifetimes of muons based on the electrons formed via muon decay. As the decay of muons to electrons and absorption into the nucleus compete, the total lifetime of the muon decreases when it is captured in an atom. As a result, the apparent lifetime of the muon is < 2.2 μs, with unique values for each element, e.g., 2.0 μs in C and 0.21 μs in Fe^10^. Therefore, identifying the atoms capturing the muons is possible by measuring their lifetimes^20,21^, i.e., elemental analysis via lifetime measurement is possible if the lifetimes are sufficiently different among the components. This method displays three main advantages. First, the muon capture probability of each element depends on the composition of the muon capturing material, and multi-elemental analysis is possible^8^. Second, the muon lifetime in a heavy element is short, and it is rapidly absorbed into the nucleus, whereas that in a lighter element is longer^10^. As a result, the electrons formed via decay of the muons captured by light elements are continuously emitted, even after all muons captured by the heavy elements have decayed. This suggests that small amounts of light elements may be identified by measuring the components with long lifetimes, even if they coexist with large amounts of heavy elements. Finally, non-destructive depth profile analysis by controlling the incident muon energy within the material is possible. As muons exhibit a sharp stopping distribution with depth in a material, depending on the incident muon energy, non-destructive, regioselective analysis is possible. Depth profile analyses in the range of several micrometers to millimeters have been performed using muonic X-ray measurements^12–14,16,19^. In addition, high-energy electrons formed via muon decay may easily exit the material from depth, rendering their detection possible without absorption by the material itself. Based on these features, when negative muons are irradiated onto a sample comprising ≥ 2 elements with different muon lifetimes, the elemental composition of the bulk material at any position (depth) may be non-destructively determined by measuring the lifetime spectrum. This is based on the electrons formed via muon decay. In this study, non-destructive identification of the C content in steel was established as a novel analytical method.

## Results

### Determination of muon arrival time

To analyze the lifetime spectrum of the muons based on the electrons formed via muon decay, the precise times of muon introduction and stoppage within the sample are crucial. As the flight velocity of the muon in the beamline changes based on the energy of the muon, the timing of muon stoppage with-in the sample changes slightly with the incident energy of the muon. As positively and negatively charged muons with the same kinetic energy exhibit the same stoppage time, the dependence of the kinetic energy on *T*_*0*_ is obtained by analyzing the lifetime spectrum of the positively charged muon irradiation of Cu. The detailed procedures used in determining *T*_*0*_ are provided in the Supporting Information S1.

### Spectral analysis

**Figure 1:**
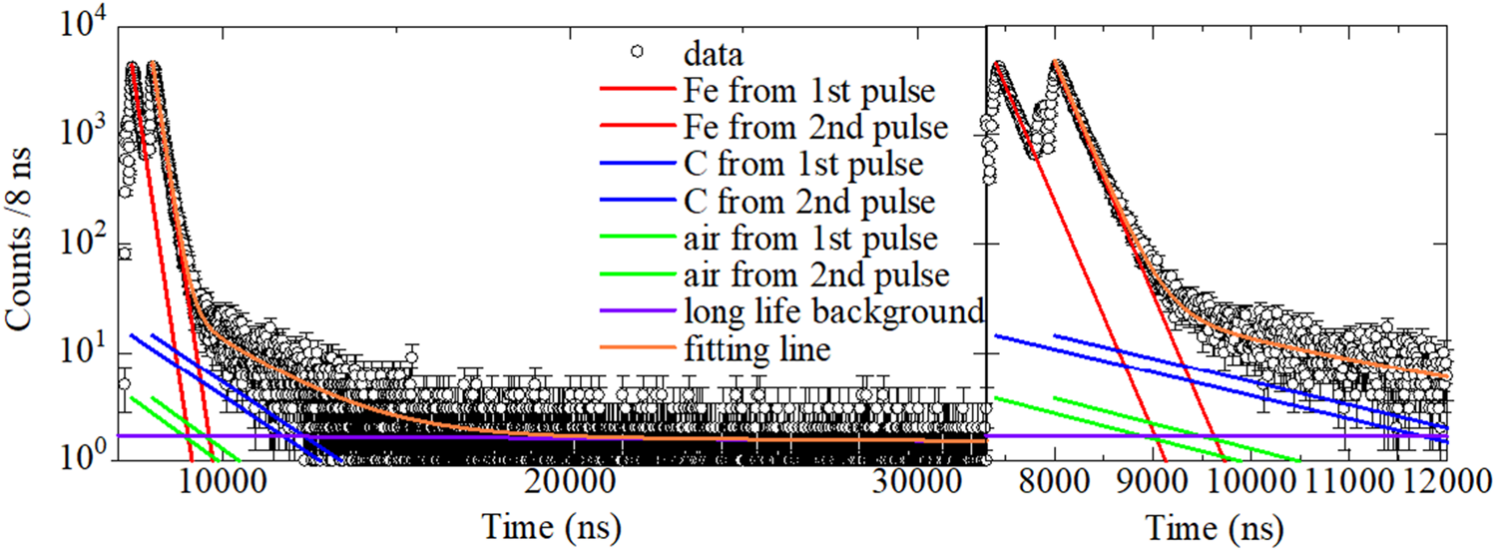
Muon lifetime spectrum of a C content of 0.42 wt% steel. (left) Entire fitted region and the (right) 7300–12,000 ns region. The fitting results including each component of Fe, C, air and background are also shown.

Figure 1 shows the lifetime spectrum of the steel containing 0.42 wt% C based on the electrons formed via muon decay in the muon irradiation study obtained from the detector set installed upstream of the beamline. The counting rate of the detected electrons is the highest at the time of arrival of the muon beam at the sample and then decreases rapidly, de-pending on the lifetime of the muon captured in each atom. The two peaks at approximately 7448 and 8048 ns correspond to the muon arrival times. These times indicate the difference between the accelerator operation time and *T*_*0*_ of a muon, and thus, no signal is observed prior to muon arrival. In this study, four components—Fe, C, air, and a background with a long lifetime—were considered in analyzing the muon lifetime spectrum obtained from the detector set in upstream (detector 1) and downstream (detector 2) along the beamline Since all measurements were carried out under atmospheric conditions, the minor component of incident muons stopped in the air. The intensity *N*(*t*) of each component (Fe, C, air and background) at time *t* is expressed by a simple exponential function: *N*(*t*) = *N*_*0*_(exp(-*λ*((*T*_*0*_ − 600) − *t*)) + exp(− *λ*(*T*_*0*_ − *t*))). *N*_*0*_ is the initial intensity at the muon beam coming and *λ* is the decay constant. In J-PARC, a double-bunch structure beam is provided, so each component has two decay functions; the first exponential component in the equation originated from the first muon beam bunch, and the second component is from the second muon beam bunch. Since the primary beam intensity of the double bunch structure is the same, the intensities of the two components are also the same.

The Fe component was analyzed as a single exponential-decaying component with a lifetime of 206.0 ns^10^. C was fitted to the component with a lifetime of 2026.3 ns^10^. The C component of 30 ppm C steel was not considered in the analysis. The third component, air, was considered as a gaseous mixture of three components, i.e., 78 mol.% N, 21 mol.% O, and 1 mol.% Ar. According to the reported muon capture probabilities^22,23^, the muon capture ratios of N, O, and Ar may be estimated as 75%, 24%, and 1%, respectively, and the respective lifetimes of each component are 1906.8, 1795.4, and 537 ns^10,24^. As the Sn beam guide is applied (for details, see section of Experimental setup and detection system), muon capture in the air occurs in only a small fraction of the air in the beam guide immediately before the muon is incident on the sample. We estimate that approximately 1/1000 of the muons irradiated on the sample stop in the air in a simulation using the Monte Carlo code GEANT4 (CERN, Meyrin, Switzerland)^25^. As the muon capture behavior in air should be identical under all sample irradiation conditions, the ratio of the number of stopped muons in air to that in the sample (the sum of Fe and C) was fixed with that obtained from pure Fe (30 ppm C steel); 0.00082 for Detector 1 and 0.00093 for Detector 2, respectively.

The origin of the last component, the background with a long lifetime, remains unknown. The intensity of these long-lived components was less than 1/1000 of the signal originating from the sample (the sum of Fe and C). The lifetime of this component is approximately 20 μs, which is much longer than the muon lifetime of 2.2 μs. These background signals may not be derived from the muon beam but from other types of radiation, such as γ-rays or neutrons, originating from the accelerator. During fitting, the lifetimes of these components for detectors 1 and 2 were fixed at 21.2 and 19.6 μs, respectively. These were determined by fitting the summed spectra for all measurements with detectors 1 and 2 in the 24,000–32,000 ns region. Notably, the contribution of the background component is negligible when analyzing C because the lifetimes are completely different.

During fitting, the 8400–32,000 ns regions of the lifetime spectra were used. A bunch of muons was assumed to exhibit the same intensity and a time interval of 600 ns. The T_0_ values of the muons on the sample at each momentum are shown in Fig. S3 in the Supporting Information. The spectra are successfully reproduced via fitting, considering the four components of C, Fe, air, and background (for pure Fe, three-component fitting was applied owing to the absence of C), and the fitting results are shown in Table 1. The components of muon capture in air are very small, and the intensities of air are < 1/1000 of those of the sample. However, we observe a small deviation between the obtained data and the fitting results around the peak region (8000–8400 ns), which may be due to two reasons: the Sn component originating from the beam guide observed close to the peak region and the finite bunch widths of the muon beams. The width of each muon bunch is approximately 100 ns and the intensity may be constant. To mitigate this, we excluded the data before the 8400 ns region, although *T*_*0*_ is approximately 8000 ns. As the lifetime of the muon captured in Sn originating from the beam guide is approximately 86 ns^10^, the Sn component decreases after the 8400 ns region to < 1/200 of the initial intensity, and this component no longer influences the results.

**Table 1:** Summary of the lifetime analyses of the samples under muon irradiation. Each intensity, except that of the component with a long lifetime, is normalized per single muon pulse. The intensities of air, except that of pure Fe, are fixed based on the ratio of the numbers of stopped muons in the air and sample (Fe + C) of pure Fe (see text).

Sample	Incident muon energy (MeV)	Detector	Intensity			
			Air	Fe	C	Long lifetime
Pure Fe	8.5	1	3.4 ± 0.2	4182 ± 66	–	2.02 ± 0.05
		2	5.6 ± 0.2	6013 ± 93	–	1.90 ± 0.06
0.42 wt% C steel	8.5	1	3.8	4550 ± 73	14.6 ± 0.5	1.71 ± 0.06
		2	5.7	6194 ± 97	18.8 ± 0.5	1.77 ± 0.06
4.46 wt% C steel	8.5	1	1.5	1743 ± 35	65.2 ± 0.5	0.40 ± 0.06
		2	0.69	725 ± 18	22.2 ± 0.3	0.64 ± 0.05
	11.6	1	1.4	1625 ± 35	59.9 ± 0.5	0.54 ± 0.05
		2	0.72	752 ± 20	30.3 ± 0.3	0.65 ± 0.05
Stacked sample	9.8	1	3.9	4760 ± 77	19.3 ± 0.5	1.84 ± 0.06
		2	6.7	7181 ± 112	27.0 ± 0.6	1.93 ± 0.06
	7.3	1	3.9	4734 ± 75	7.4 ± 0.4	1.80 ± 0.05
		2	6.3	6757 ± 104	9.3 ± 0.5	1.74 ± 0.06
	4.9	1	4.0	4811 ± 77	44.1 ± 0.6	1.72 ± 0.07
		2	6.3	6732 ± 104	55.8 ± 0.7	1.77 ± 0.07

## Discussion

**Figure 2:**
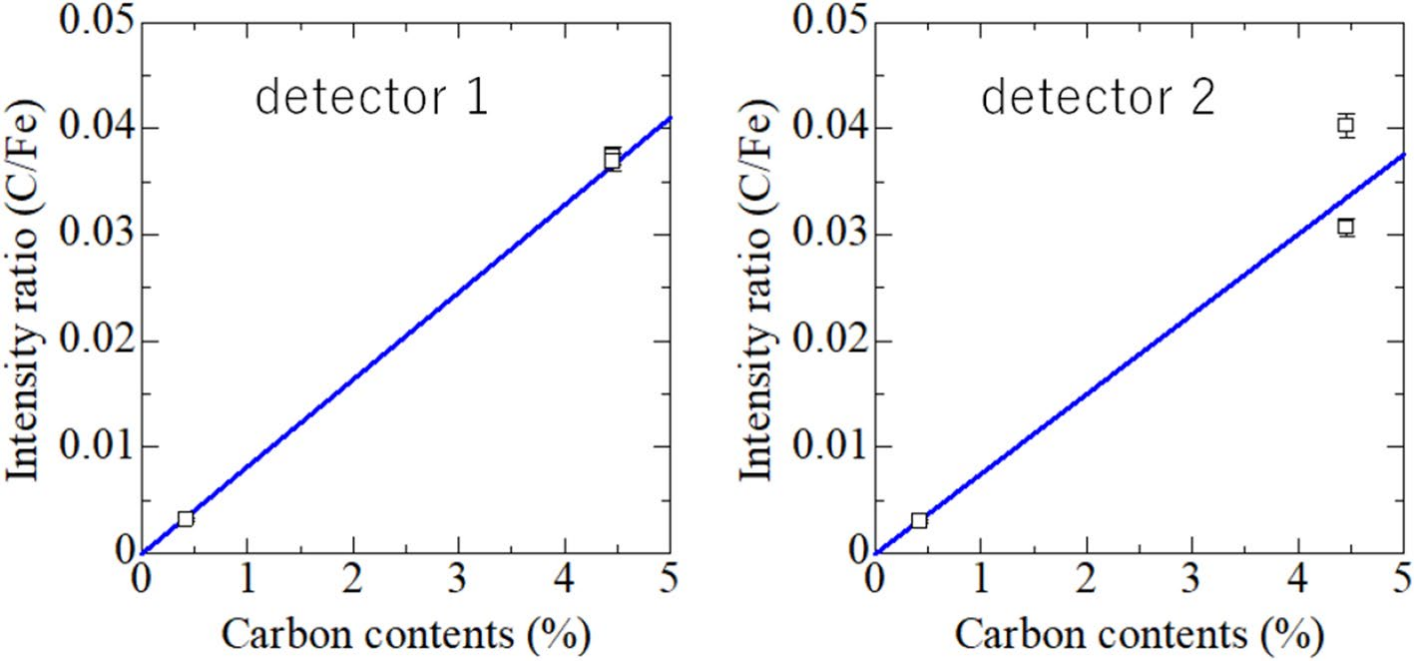
Relationship between the C contents in steel and C/Fe signal intensity ratios obtained by measuring the muon lifetimes using detectors 1 and 2 together with fitting lines by a weighted least square fitting method.

In quantitative analysis, the relationship between the signal intensity of C obtained via measuring the muon lifetimes and the elemental composition of the sample obtained via chemical methods should be determined. Figure 2 shows the relationship between the C content on the horizontal axis and the C/Fe signal intensity ratio obtained via muon irradiation on the vertical axis.

For the results obtained using detectors 1 and 2, the relationships are reproduced well by linear lines. The slopes of these lines for detectors 1 and 2 are 0.00820 ± 0.00019 and 0.00750 ± 0.00062, respectively. Using detector 2, the standard steel with 4.46 wt% C exhibits different values at two incident energies (8.5 and 11.6 MeV), because the sample is very thick (thickness: 20 mm), and a large proportion of the emitted electrons is absorbed by the sample before reaching the detector. As shown in Table 1, the counts of this sample are considerably lower than those of the other samples. This problem is not observed when using detector 1, which detects electrons from the upstream side of the muon beam. Although the muon stoppage depths of 0.95 and 1.63 mm differ at the two incident muon energies, this difference hardly affects electron absorption and does not influence the results.

Based on these relationships, determining the elemental composition using the signal intensity obtained via muon irradiation is possible. The slope of the calibration line is almost 0.01, and thus, the signal intensity and elemental composition ratios are almost identical. Hence, the capture probabilities for the elements are almost identical, although the atomic numbers of C and Fe differ significantly. This is consistent with previous studies that report that the muon capture probability of each constituent element in an alloy is constant over a wide concentration range^26^.

The detection limit was estimated using a calibration curve. Distinguishing between the air and C components is challenging owing to their similar lifetimes. Consequently, the detection limit of C is determined by on the uncertainty due to the air component. Based on the results for pure Fe, when three times the error of the air component was applied as the detection limit, the detection limits of C/Fe are estimated to 0.00013 and 0.00011 for detectors 1 and 2, respectively. Using calibration curves, these limits correspond to 160 and 140 ppm. Owing to these detection limits, the quantification of C in steel containing 30 ppm C (regarded as pure Fe in this study) is unsuccessful. The detection limit should decrease in the future due to improvements in the experimental setup, e.g., the removal of the air around the sample and preparation of a vacuum chamber.

To evaluate the performance of this method, muon irradiation studies of the stacked sample with three steel plates with thicknesses of 0.5 mm and C concentrations of 1.03, 0.20, and 0.51 wt% were conducted. By controlling the incident muon energies, the muon stoppage depth could be adjusted, i.e., a non-destructive depth profile analysis was possible. When muons were introduced into the sample at an energy of 4.9 MeV, the average depth of muon stoppage was 0.35 mm from the surface. Assuming a muon energy width of 5%, the distribution was evaluated as 0.30–0.42 mm. Using 7.3 MeV muons, the average depth of muon stoppage was 0.73 mm, with a distribution of 0.60–0.86 mm, i.e., the muons passed completely through the first layer of stacked steel and stopped in the second layer. Using 9.8 MeV muons, they stopped in the third layer. The muon stoppage region corresponded to 1.02–1.42 mm, with an average depth of muon stoppage of 1.21 mm. The muon stoppage depth of each incident muon energy was estimated using the Stopping and Range of Ions in Matter (SRIM) code (IBM, Armonk, NY, USA)^27^.

As shown in Table 1, different results are obtained at each incident muon energy, i.e., muon stoppage position. The C content at each incident muon energy was determined using the calibration curve, and the results are shown in Table 2. As the self-absorption effects of the two sets of detectors differ, the C:Fe ratios are calculated independently for these detectors. The weighted average values of the two detectors are then adopted because the results are consistent. The results of this study reproduce the C concentration in each steel layer, as determined via chemical analysis. These analytical values are obtained non-destructively, and depth-profile quantification is realized by simply changing the incident energies of the muons. Using muon lifetime measurements, we successfully determined the sub-percent C content in steel without sample destruction.

**Table 2:** Comparison of the C contents determined using the muon and chemical analyses.

Incident muon energy (MeV)	Estimated muon stoppage depth (mm)	C contents determined via muon analysis (wt%)			C content determined via chemical analysis (wt%)
		Detector 1	Detector 2	Average	
9.8	1.21	0.495 ± 0.020	0.502 ± 0.044	0.50 ± 0.02	0.51
7.3	0.73	0.192 ± 0.013	0.183 ± 0.018	0.19 ± 0.01	0.20
4.9	0.35	1.117 ± 0.036	1.104 ± 0.094	1.12 ± 0.03	1.03

## Conclusions

We clarified that the sub-percent C content in steel could be quantified by measuring the muon lifetimes based on measurements of decay electrons emitted after muon irradiation of the steel. This method can be performed simultaneously with the elemental analysis method by measuring muonic X-rays. This analytical method was non-destructive, with a low C detection limit of 140 ppm. Such superior properties were due to (1) the extremely high penetration of the decay electrons, which are highly sensitive to light elements, and (2) the rapid decay of electrons originating from muons captured in Fe atoms, whereas those originating from muons captured in C may be detected after those originating from Fe are fully decayed under low background conditions. The detection limit was mainly derived from muon stoppage in the air around the sample and may thus be improved by preparing a chamber without air around the sample. The calibration curve prepared using the standards displayed a good linearity, enabling quantitative analysis. A stacked sample comprising three types of steel with different C contents was irradiated with muons with different incident energies, and depth profiling of the C content was realized. This method may be used in analyzing the interior of a sample without destruction and does not require sample pretreatment to remove surface contamination. Furthermore, because of its non-destructive properties, this method may be applied in analyzing valuable samples, such as steel archaeological artifacts.

## Materials and methods

### Muon facility

The muon study was conducted in the D1 experimental area of the Muon Experimental Facility (MUSE) of the Materials and Life Science Facility of the Japan Proton Accelerator Research Complex (J-PARC, Tokai, Japan). MUSE provides the highest-pulsed muon beam in the world^28^, which enables precise muon studies. The muon beam was generated by bombarding a graphite muon production target with high-energy protons accelerated to 3 GeV by a synchrotron accelerator, and muons of various energies were generated simultaneously. The energies of the extracted muons could be controlled using the electromagnet system of the beamline, and a muon beam with a specific energy and an energy dispersion of 5% could be obtained at the beamline exit. A muon beam with a 25 Hz double-bunch structure was supplied, according to the characteristics of the proton synchrotron accelerator. The time interval between the double bunches was 600 ns, and the width of each bunch was approximately 100 ns. Pb and W–Ni alloy collimators with inner diameters of 20 mm were installed at the exit of the beamline for beam shaping. The muon beam passed through the vacuum window at the end of the beam-line and was extracted in the air.

### Experimental setup and detection system

**Figure 3:**
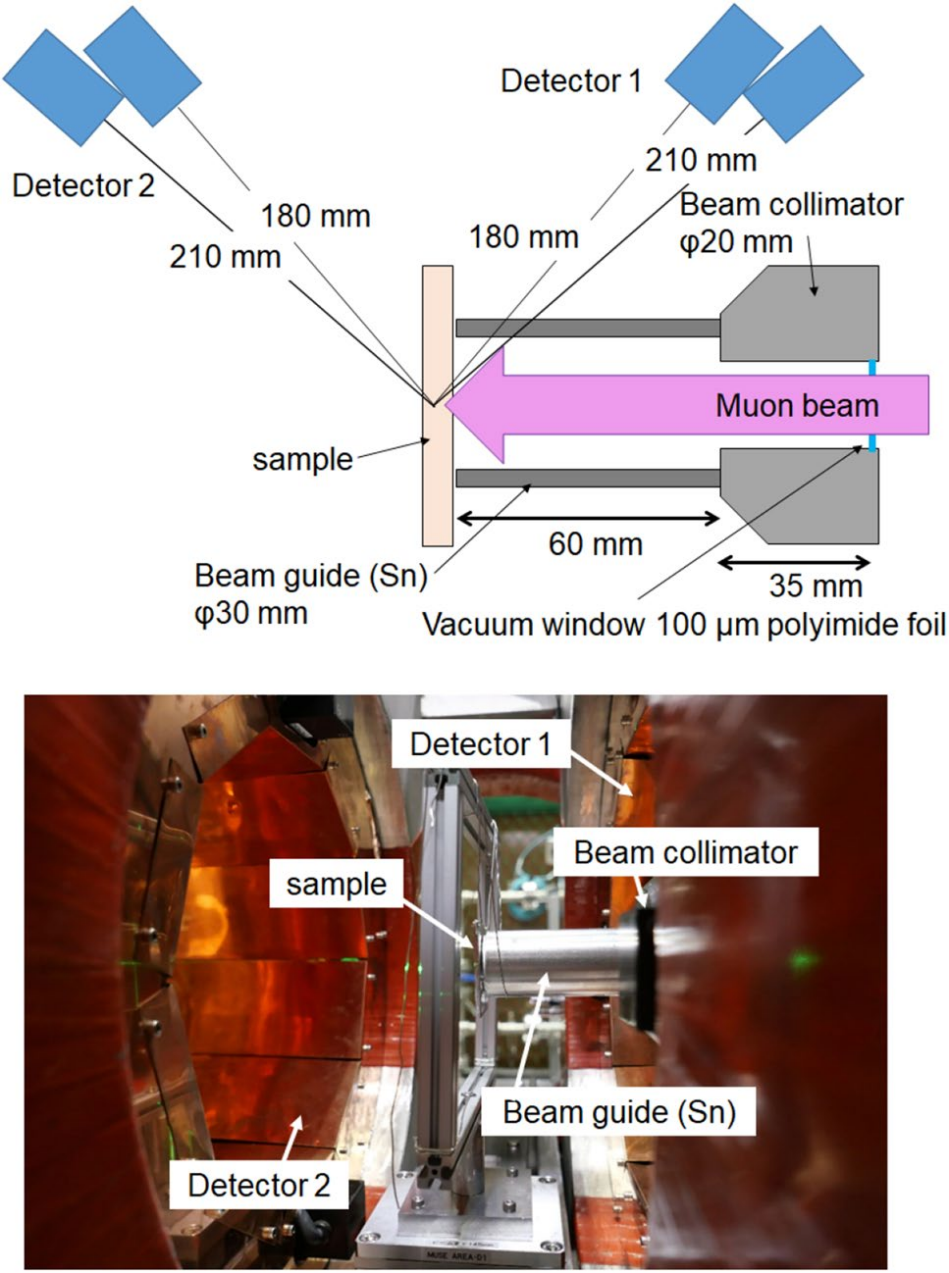
Schematic diagram (top) and image (bottom) of the experimental setup. Electrons from muon decay were measured by two detector sets (Detector 1 and 2) placed upstream and downstream of the sample. The experiment was conducted under atmospheric conditions. A Sn-duct was set between the beamline exit and the sample to eliminate scattered muon.

A steel sample was installed 9.5 cm downstream from the beamline exit, as shown in Fig. 3. Electrons emitted after the muons stopped in the sample were measured using plastic scintillation counters. After extracting the beamline into the air, the muon beam spread along the flight distance. The muons that did not stop in the sample stopped in the surrounding air and decayed to electrons, generating a background signal. To eliminate such background components, a Sn duct (beam guide) with a thickness and length and an inner diameter of 2 mm, 6 cm, and 30 mm, respectively, was installed between the sample and exit of the beamline. The lifetime of a muon captured in Sn is much shorter than those of muons captured in Fe and C, which are the constituent elements of steel. An electron detection system denoted KALLIOPE, which covered a solid angle of 23%, was equipped at the D1 experimental area at MUSE^29^. The detection system comprised two sets of detectors arranged on the circumference at the incident (detector 1) and downstream (detector 2) sides of the muon beam. Each set of detectors was divided into 640 small detectors, which each comprised two stacked plastic scintillation counters. Such a stacked detector was highly effective in reducing the background signal, and a true detected signal was counted only when both plastic scintillators detected the electron simultaneously, i.e., when coincident signals were obtained from the two detectors. This system was already developed for use in muon spin rotation studies^30^ and it could also be used in elemental analysis via muon lifetime measurement in this study. The muon lifetime spectrum could be obtained by determining the difference between the accelerator operation and muon detection times.

### Muon irradiation study

Three thick steel plates and one stack of three types of thin steel plates were prepared as samples for use in muon analysis. The thick plates were used as standard samples, and the stacked sample was used to verify the depth profile analysis. The compositions of the samples were obtained using chemical analyses. The three thick steel samples exhibited C contents of 30 ppm (0.003 wt%, considered as pure Fe) and 0.42 and 4.46 wt%, and the sample sizes are shown in Table 3. At J-PARC, the muon beam intensity becomes higher with increasing the muon energy^28^, so we set a relatively long irradiation time in low momentum conditions. The layers of the stacked sample displayed thicknesses of 0.5 mm, with C contents of 1.03, 0.20 and 0.51 wt%. The samples were larger than the inner diameter of the muon beam guide comprised of Sn (30 mm). The muons exited the beamline and stopped in the sample, except several muons that stopped in the Sn beam guide or the air within the beam guide.

**Table 3:** Summary of sample data and muon irradiation conditions of each sample. The muon irradiation times are calculated using the number of irradiated muon pulses, based on accelerator cycling of 25 Hz.

Sample	Size (mm)	Incident muon energy (MeV)	Number of irradiated muon pulses	Muon irradiation time (h)
30 ppm C steel (pure Fe)	50 × 50 × 1.5	8.5	190 621	2.1
0.42 wt% C steel	φ37 × 2.0	8.5	187 829	2.1
4.46 wt% C steel	φ30 × 20	8.5	81 264	0.9
		11.6	46 353	0.5
Stacked sample (1.03 wt%, 0.20 wt%, and 0.51 wt% C)	50 × 50 × 1.5 (thickness of each layer: 0.5 mm)	9.8	161 764	1.8
		7.3	256 441	2.8
		4.9	563 337	6.3

The muon irradiation times of these samples were 0.5–6.3 h, and the irradiation conditions are shown in Table 3. The incident energies of the muons were set to 4.9–11.6 MeV (32.4–50.8 MeV/c in terms of muon momentum), corresponding to a muon stoppage depth of 0.35–1.63 mm in pure Fe^26^. Further-more, to determine the arrival times (*T*_*0*_) of the muons on the sample, positively charged muon irradiation of pure Cu was performed under a magnetic field of 123 G applied in the direction perpendicular to the beam (transverse magnetic field condition). Details of the positive muon study are provided in the Supporting Information S1.

### Supplementary Information

Supplementary Information 1.

Supplementary Information 2.

## Data Availability

All data generated during this study are included in this published supplementary information file.
