# Supplementary material for: Development of a non-destructive depth-selective quantification method for sub-percent carbon contents in steel using negative muon lifetime analysis
Source: Sci Rep. 2024 Jan 20;14:1797. doi: 10.1038/s41598-024-52255-5 (PMC10799958; doi:10.1038/s41598-024-52255-5)
Supplement: Supplementary file 2 — Supplementary Information 2. [file 41598_2024_52255_MOESM2_ESM.docx]

Supporting information

for

Development of a non-destructive depth-selective quantification method for sub-percent carbon contents in steel using negative muon lifetime analysis

Kazuhiko Ninomiya^*1,2^, Michael Kenya Kubo^3^, Makoto Inagaki^4^, Go Yoshida^5^, I-Huan Chiu^1^, Takuto Kudo^2^, Shunsuke Asari^2^, Sawako Sentoku^3^, Soshi Takeshita^6^, Koichiro Shimomura^6^, Naritoshi Kawamura^6^, Patrick Strasser^6^, Yasuhiro Miyake^6^, Takashi U. Ito^7^, Wataru Higemoto^7,8^, and Tsutomu Saito^9^

1 Institute for Radiation Sciences, Osaka University, 1-1, Machikaneyama, Toyonaka, Osaka 560-0043, Japan

2 Graduate School of Science, Osaka University, 1-1, Machikaneyama, Toyonaka, Osaka 560-0043, Japan

3 Division of Natural Sciences, International Christian University, 3-10-2, Osawa, Mitaka, Tokyo 181-8585, Japan

4 Institute for Integrated Radiation and Nuclear Science, Kyoto University, Asashiro-nishi, Kumatori, Osaka 590-0494, Japan

5 Radiation Science Center, High Energy Accelerator Research Organization (KEK), 1-1, Oho, Tsukuba, Ibaraki 315-0801, Japan

6 Muon Science Laboratory, Institute of Materials Structure Science, High Energy Accelerator Research Organization (KEK), 1-1, Oho, Tsukuba, Ibaraki 315-0801, Japan

7 Advanced Science Research Center, Japan Atomic Energy Agency, 2-4, Shirakata, Tokai, Ibaraki 319-1195, Japan

8 Department of Physics, Tokyo Institute of Technology, 2-12-1, Ookayama, Meguro, Tokyo 152-8550, Japan

9 National Museum of Japanese History, 117 Jonai-cho, Sakura, Chiba 285-8502, Japan

* E-mail: ninomiya@rirc.osaka-u.ac.jp

**Supporting Information**

**Table of contents**

**Determination of the muon arrival time (*T_0_*) using the muon spin rotation (μ^+^SR) method**

**Supplementary figures**

**Determination of the muon arrival time (*T_0_*) using the muon spin rotation (μ^+^SR) method**

**Purpose and principle**

To determine the intensity of each component using the muon lifetime spectrum, determining the precise initiation time of the lifetime spectrum, which is the *T_0_* at each incident muon energy, is necessary.

*T_0_* may be determined via the investigation of the spin rotation behavior of the muon under a transverse magnetic field using the μ^+^SR method^1^. Negatively charged muons were used in the lifetime measurements in the main part of this study, but positively charged muons were ideal for this purpose. Unlike negatively charged muons, positively charged muons are not absorbed by the nucleus, and thus, their lifetimes are 2.2 μs in any material. Although positively charged muons emit positrons instead of electrons during decay, we may use the same detection system in the D1 experimental area for electron measurement^2^. The detection system was originally prepared for use in μ^+^SR studies employing positively charged muons. A positive muon may be extracted by adjusting the electromagnetic system of muon transportation at the beamline at the Japan Proton Accelerator Research Complex (J-PARC, Tokai, Japan) in the same manner as that used in extracting negative muons. A positively charged muon is transported through the beamline in the same manner as that of a negatively charged muon, thus similarly arriving on the sample.

Muons generally emit positrons in the spin direction during muon decay, and thus, the muon spin behavior may be investigated by measuring the emission direction. The muons extracted from the beamline exhibited the same spin direction, which was the opposite of that of muon flight. When the external magnetic field is perpendicular to the spin direction (transverse magnetic field), the muon spin precesses in the direction of the external magnetic field, i.e., the direction of positron emission, which depends on the spin direction, is also time-dependent. In addition, positively charged muons do not lose their spin polarization in Cu, and thus, spin rotation may be observed with a high efficiency. The time at which the rotation commenced corresponded to *T_0_*. As the muon transportation beamline was 12 m in length, *T_0_* at the target varied, depending on the muon kinetic energy, and thus, we observed the spin rotation behavior at each incident energy.

**Experimental method**

The study was performed using the same setup as that used in negatively charged muon irradiation (Figure 1). A Cu plate (99.996%) with a size and thickness of 50 × 50 and 2 mm, respectively, was prepared as the muon irradiation sample. At the sample position, a transverse magnetic field of 123 G was applied perpendicular to the muon spin direction. This magnetic field corresponded to the muon motion during the time interval between pulses of the muon double-bunch beam structure (600 ns) ^3^. The energies of the incident muons were adjusted to 4.9–11.6 MeV, and the duration of muon irradiation at each incident muon energy was tens of minutes. The positrons emitted by muon decay were measured using two sets of detectors equipped at the up- and downstream sides of the sample (detectors 1 and 2).

**Analysis and results**

Figure S1 shows the raw spectra obtained using detectors 1 and 2, and spin rotation induced by the external magnetic field is clearly observed. In addition, as shown in Figure S2, the asymmetry of the count obtained using the two detectors displays oscillations, depending on the magnetic field. The asymmetry *A(t)* may be obtained using the following equation:

$A\left( t \right)=\frac{N_{2}\left( t \right)-N_{1}\left( t \right)}{N_{2}\left( t \right)+N_{1}\left( t \right)}$ (S1)

where *N_1_(t)* and *N_2_(t)* are the numbers of counts measured using detectors 1 and 2 at time *t*. As all spins are aligned when the muon is introduced into the sample, the time dependence of *A(t)* reveals oscillations depending on the magnetic field. *T_0_* of each incident muon energy was determined by fitting *A(t)* to a cosine curve. The analytical method is commonly used in μ^+^SR studies and the details are described in the literature^1^.

Figure S3 shows the dependence of the muon incidence time on the muon energy, as determined via the analysis. The horizontal axis corresponds to the deviation of the muon detection time from the accelerator operation time, e.g., at an incident muon energy of 4.9 MeV, the average second pulse time is 8048 ns.

**References**

1. Blundell, S. J., Renzi, R. D., Lancaster, T. & Pratt, F. L. Muon Spectroscopy: An Introduction, Oxford University Press (2022).

2. Kojima, K. M. *et al.* New μSR Spectrometer at J-PARC MUSE Based on Kalliope Detectors. *J. Phys. Conf. Ser.* **551**, 012063 (2014).

3. Miyake, Y. *et al.* J-PARC Muon Source, MUSE. *Nucl. Instrum. Meth. Phys. Res. A.* **600**, 22–24 (2009).

**Figure Captions**

Figure S1: Lifetime spectra of the positrons in the Cu sample obtained using an incident muon energy of 8.5 MeV, as measured using detectors 1 and 2.

Figure S2: Time dependence of the asymmetry obtained via positive muon irradiation of the Cu sample with an incident muon energy of 4.6 MeV and the fitting line obtained using a cosine curve.

Figure S3: Dependence of *T_0_* of the second bunch of the muon beam on the incident muon energy.

Figure S1:

Figure S2:

Figure S3:
